# Molecular Imaging-Guided Theranostics and Personalized Medicine

**DOI:** 10.1155/2013/859453

**Published:** 2013-10-24

**Authors:** David J. Yang, Fong Y. Tsai, Tomio Inoue, Mei-Hsiu Liao, Fan-Lin Kong, Shaoli Song

**Affiliations:** ^1^Diagnostic Imaging, The University of Texas MD Anderson Cancer Center, Houston, TX 77030, USA; ^2^Taipei Medical University, Taipei 11031, Taiwan; ^3^University of California at Irvine, Orange, CA 92868, USA; ^4^Department of Radiology, Yokohama City University School of Medicine, 3-9 Fukuura Kanazawa-ku, Yokohama 236-0004, Japan; ^5^Radiopharmaceuticals Production and Marketing Center, Institute of Nuclear Energy Research, Atomic Energy Council, Taoyuan 32546, Taiwan; ^6^Department of Cancer Systems Imaging, The University of Texas MD Anderson Cancer Center, Houston, TX 77030, USA; ^7^Renji Hospital, School of Medicine, Shanghai Jiao Tong University, Shanghai 200127, China

Molecular imaging science has been focused on imaging guidance in the areas of targeting epigenetic abnormalities and tumor microenvironment in overcoming resistance in cancers. The use of image-guided technologies to select patient for personalized therapy and to monitor therapeutic outcomes is the focus of this special issue. For instance, mutations in the kinase domain of epidermal growth factor receptor (EGFR) have been associated with clinical responsiveness using tyrosine kinase inhibitors for nonsmall cell lung cancer (NSCLC). S. H.-H. Yeh et al. reported the feasibility of using morpholino-[I-124]IPQA as an in vivo PET imaging probe for the expression of different EGFR mutants in NSCLC. Their micro-PET imaging along with biologic validation indicated that [I-124]IPQA derivative might be a useful probe in selecting the patients with NSCLC for tyrosine kinase inhibitor therapy.

Molecular imaging enables the comprehensive characterization of therapeutic intervention and can be used in preclinical studies, pharmacokinetic (microdosing) studies, dose-finding studies, and proof-of-concept studies. F. Guerriero et al. reported the most adequate timing for imaging and kidney dosimetry in Lu-177 and Y-90 labeled DOTATATE and DOTATOC by SPECT in 1-2 scans.

Fluorodeoxyglucose (FDG) is a gold standard glycolytic agent in nuclear imaging. For instance, A. Bunevicius et al. suggested that FDG PET might serve as an alternative and noninvasive tool to MRI and CT for the management of acute stroke patients. However, FDG has poor differentiation between inflammation/infection and tumor recurrence. To characterize cancers, new tracers have been focused on target expressions, pathway directed therapies, and cell functions in the intact organism. F.-L. Kong et al. reported promising theranostic radiotracers beyond FDG that are currently under preclinical development and clinic management in lymphoma. I.-H. Shih et al. also reported the alternative of using F-18 labeled alpha-methyltyrosine to assess amino acid transporter systems in mesothelioma models.

Topics covered in this special issue are advances in molecular imaging both in radioactive and nonradioactive applications in preclinical drug discovery, drug development, drug delivery, pharmacokinetics and pharmacodynamics, and differential diagnosis. For nonradioactive molecular imaging technology, F. C. Wong et al. reported photo affinity labeling using tissue-penetrating radiation (X-ray or gamma rays), which could overcome the tissue attenuation and irreversibly label membrane receptor proteins. They described that X-ray and gamma rays could induce affinity labeling of membrane receptors in a manner similar to UV with photo reactive ligands of the dopamine transporters, D2 dopamine receptors, and peripheral benzodiazepine receptors. P.-C. Chu et al. reported that the brain tumor conditions on the distribution and dynamics of small molecule leakage into targeted regions of the brain could be influenced by focused ultrasound (FUS)-BBB opening. Their findings indicated that FUS-BBB opening might have the most significant permeability-enhancing effect on tumor peripheral. Their report provides useful information toward designing an optimized FUS-BBB opening strategy to deliver small-molecule therapeutic agents into brain tumors.

MRI has been proven to be a valuable tool to provide important information facilitating individualized image-guided treatment and personalized management for cancers. J.-H. Chen and M.-Y. Su reviewed the use of different MR imaging methods, including dynamic contrast-enhanced MRI proton MR spectroscopy, and diffusion-weighted MRI, to monitor and evaluate the treatment response. They also described how the changes of parameters measured at an early time after initiation of a drug regimen could predict final treatment outcome. H.-W. Kao et al. also reviewed advanced MR imaging techniques including cellularity, invasiveness, mitotic activity, angiogenesis, and necrosis in gliomas. Molecular imaging with MRI also permits mapping and measuring the rate of physiological, biochemical, and molecular process with the use of appropriate kinetic models. For instance, T. Y. Siow et al. described their MRI findings with increased nNOS activity in brain cortex and striatum after 1-methyl-4-phenyl-1,2,3,6-tetrahydropyridine (MPTP) treatment. They concluded that the transient changes in hyperperfusion state in cerebral blood flow in the cortex and striatum might be an early indicator of neuronal inflammation.

T.-L. Yang et al. compared the diagnostic performance of digital breast tomosynthesis (DBT) and digital mammography (DM) for breast cancers. They concluded that adjunctive DBT provided exquisite information for mass lesion, focal asymmetry, and/or architecture distortion which could improve the diagnostic performance in mammography. Y.-C. Liu et al. reported the validation of the clinical significance of coronary artery calcium score (CACS) in predicting coronary artery disease (CAD) and cardiac events using a 64-slice coronary CT angiography. They concluded that CACS was significantly correlated with CAD and cardiac events.

The emergence of flat-detector X-ray angiography in conjunction with contrast medium injection and specialized reconstruction algorithms can provide not only high-quality and high-resolution CT-like images but also functional information. This improvement in imaging technology allows quantitative assessment of intracranial hemodynamics and subsequently in the same imaging session. S.-C. Hung et al. described the recent developments in the field of flat-detector imaging and shared their experience of applying this technology in neurovascular disorders such as acute ischemic stroke, cerebral aneurysm, and steno occlusive carotid diseases.

*David J. Yang* *Fong Y. Tsai* *Tomio Inoue* *Mei-Hsiu Liao* *Fan-Lin Kong* *Shaoli Song*

